# Subclade 2.2.1-Specific Human Monoclonal Antibodies That Recognize an Epitope in Antigenic Site A of Influenza A(H5) Virus HA Detected between 2015 and 2018

**DOI:** 10.3390/v11040321

**Published:** 2019-04-02

**Authors:** Moe Okuda, Seiya Yamayoshi, Ryuta Uraki, Mutsumi Ito, Taiki Hamabata, Yoshihiro Kawaoka

**Affiliations:** 1 Division of Virology, Department of Microbiology and Immunology, Institute of Medical Science, University of Tokyo, Minato-ku, Tokyo 108-8639, Japan; 1923890575@edu.k.u-tokyo.ac.jp (M.O.); r-uraki@med.nagoya-cu.ac.jp (R.U.); ito-mu@ims.u-tokyo.ac.jp (M.I.); hamabata@ims.u-tokyo.ac.jp (T.H.); 2 Department of Special Pathogens, International Research Center for Infectious Diseases, Institute of Medical Science, University of Tokyo, Minato-ku, Tokyo 108-8639, Japan; 3 Department of Pathobiological Science, School of Veterinary Medicine, University of Wisconsin-Madison, Madison, Wisconsin 53706, USA

**Keywords:** Influenza A virus, H5-HA, human monoclonal antibody, escape mutant virus

## Abstract

Highly pathogenic avian H5 influenza viruses persist among poultry and wild birds throughout the world. They sometimes cause interspecies transmission between avian and mammalian hosts. H5 viruses possessing the HA of subclade 2.3.4.4, 2.3.2.1, 2.2.1, or 7.2 were detected between 2015 and 2018. To understand the neutralizing epitopes of H5-HA, we characterized 15 human monoclonal antibodies (mAbs) against the HA of H5 viruses, which were obtained from volunteers who received the H5N1 vaccine that contains a subclade 2.2.1 or 2.1.3.2 virus as an antigen. Twelve mAbs were specific for the HA of subclade 2.2.1, two mAbs were specific for the HA of subclade 2.1.3.2, and one mAb was specific for the HA of both. Of the 15 mAbs analyzed, nine, which were specific for the HA of subclade 2.2.1, and shared the VH and VL genes, possessed hemagglutination inhibition and neutralizing activities, whereas the others did not. A single amino acid substitution or insertion at positions 144–147 in antigenic site A conferred resistance against these nine mAbs to the subclade 2.2.1 viruses. The amino acids at positions 144–147 are highly conserved among subclade 2.2.1, but differ from those of other subclades. These results show that the neutralizing epitope including amino acids at positions 144–147 is targeted by human antibodies, and plays a role in the antigenic difference between subclade 2.2.1 and other subclades.

## 1. Introduction

The first human case of infection with a highly pathogenic avian H5N1 influenza virus was reported from Hong Kong in 1997 [1]. To date, 860 cases including 454 deaths have been recorded in 16 countries, mainly in Asia and Africa [2]. All of these cases were caused by viruses possessing H5-HA that originated from A/goose/Guangdong/1/1996 [3]. This lineage of viruses is classified into 10 clades, plus many subclades based on HA sequence similarity [3]. After 2015, H5 viruses classified into subclades 2.3.4.4, 2.3.2.1, 2.2.1 and 7.2 are mainly detected in Southeast Asia, Europe, and North America, Indonesia and Bangladesh, Egypt and Israel, and China, respectively [4,5,6]. In each of these regions, viruses continue to evolve independently.

The reassortant H5 viruses possessing HA derived from subclade 2.3.4.4, and NA from viruses other than the N1 subtype appeared, and have been spread throughout the world by migratory birds [7]. One such reassortant, the H5N6 viruses, caused 14 human cases, indicating that we must pay attention to these reassortant viruses [8].

Twenty-two human monoclonal antibodies (mAbs) that specifically bind to H5-HA have been reported (Table 1) [9,10,11,12,13,14,15,16,17,18]. Ten clones (H5.3, H5.2, H5.9, H5.13, H5.31, H5.16, H5.22, H5.24, H5.36, and H5.7), which were obtained from humans who were vaccinated with a virus classified in clade 1, bound to the H5-HA of clade 1, but did not bind to the H5-HA of the subclade 2.1.3.2 [11]. Clone H5.3 recognized epitopes in antigenic site A of H5-HA [10,11]. The other 12 clones that were obtained from patients who were infected with an H5 virus classified in clade 1 or 2.3.4 showed neutralization activity against several H5 viruses classified in different subclades [9,10,11,12,13,14,15,16,17,18]. The epitopes of these clones mapped to various regions [11,12,13,14,15,16,17]. These human mAbs are useful for antigenic analyses of HA between subclades, or within a subclade, because sequence comparisons would not reveal antigenic variation. However, the epitopes on H5-HA have not been fully determined until now, because of the limited number of available human mAbs against H5-HA.

Previously, we obtained human broadly reactive mAbs from healthy human volunteers who received the H5N1 vaccine that contains the inactivated, adjuvanted whole-virion of A/Egypt/N03072/2010 (subclade 2.2.1) or A/Indonesia/5/2005 (subclade 2.1.3.2) [19]. In the process, we also found 15 human mAbs that specifically recognized H5-HA. Here, we characterized these mAbs to better understand the antigenicity of H5-HA.

## 2. Materials and Methods

### 2.1. Ethics Statement

Human blood was collected by the following protocols approved by the Research Ethics Review Committee of the Institute of Medical Science, at the University of Tokyo (25-58-1205 and 29-72-A0322). Informed consent was obtained from all participants.

### 2.2. Cells

Human embryonic kidney 293T cells were maintained in Dulbecco’s modified Eagle’s medium (DMEM) containing 10% Fetal Calf Serum (FCS) and 1% Penicillin/Streptomycin. Madin-Darby canine kidney (MDCK) cells were maintained in Eagle’s minimal essential medium (MEM) containing 5% newborn calf serum (NCS) and 1% Penicillin/Streptomycin. These cells were incubated at 37 °C under 5% CO_2_. Cloned hybridomas expressing human antibodies were maintained in DMEM containing 15% FCS and 1% Penicillin/Streptomycin. Expi293 cells (Grand Island Biological Company (Gibco), Grand Island, New York State, USA) maintained in Expi293 expression medium (Thermo Fisher Scientific, Waltham, Massachusetts, U.S.) under serum-free conditions, were incubated on an orbital shaker platform rotating at 125 rpm at 37 °C under 8% CO_2_.

### 2.3. Viruses

H5 subtype viruses [A/gyrfalcon/Washington/41088-6/2014 (Washington; subclade 2.3.4.4, H5N8, EPI860648), A/chicken/Czech Republic/1688-171/2017 (Czech; subclade 2.3.4.4, H5N8, EPI1021136), A/chicken/Ghana/15VIR5480-7/2015 (Ghana; subclade 2.3.2.1, H5N1, EPI806495), A/duck/Menia/1543S/2015 (Menia; subclade 2.2.1, H5N1, EPI965960), and A/chicken/Wenzhou/HAYXLG03/2015 (Wenzhou; subclade 7.2, H5N2, EPI682905)] possessing conserved amino acid sequences within each subclade were selected as a representative isolate of each subclade. Attenuated nucleotide sequences of HA segments derived from these five isolates were synthesized and cloned into pHH21 for expressing vRNA. Based on the wild-type HA sequence of the Menia virus, the HA plasmid possessing a single amino acid mutation was generated by using the standard polymerase chain reaction (PCR) technique. The pHH21 plasmid encoding the attenuated wild-type or mutant HA segment, together with the other seven segments of a high-yield mutant of A/Puerto Rico/8/34 (H1N1 subtype) (HY-PR8) [20], was used to rescue the reassortant viruses [21]. At 48 h post-transfection, the culture supernatant containing the viruses was collected and then inoculated into the choriallantoic cavity of 10-day-old chicken eggs. After 72 h of incubation at 37 °C, the choriallantoic fluid was collected as a stock virus solution, and titrated by the use of a plaque assay, HA assay, and TCID_50_ (50% tissue culture infectious doses) values in MDCK cells.

### 2.4. Screening of Hybridomas Expressing Human Monoclonal Antibodies (mAbs)

The hybridomas used in this study were obtained previously [19] by fusion of peripheral blood mononuclear cells (PBMCs), which were isolated from healthy volunteers vaccinated with the H5N1 vaccine [A/Egypt/N03072/2009 (subclade 2.2.1, H5N1) or A/Indonesia/5/2005 (subclade 2.1.3.2, H5N1)], with SPYMEG cells (MBL) [22]. In this study, 15 hybridomas producing antibodies that specifically bind to H5-HA, were used.

### 2.5. Construction and Expression of Human mAbs

Total RNA was extracted from the hybridomas and reverse transcribed. The target sequences were amplified by PCR using primers targeted to the variable region of the heavy chain and the light chain of the human antibody. The sequences of the heavy and light chains were cloned into the pEHX1.1 vector and pELX2.2 vector, respectively [19,23,24]. A plasmid encoding both the heavy and light chains was constructed and transfected into Expi293 cells by using Expi Fectamine 293 (Thermo Fisher Scientific, Waltham, MA, USA) according to the manufacturer’s protocol.

At four days post-transfection, the human antibodies in the culture media were purified by using a Hi Trap rProtein A FF column (GE Healthcare, Chicago, IL, USA), and the automated chromatography system AKTA pure 25 (GE Healthcare, Chicago, IL, USA). The concentration of the purified antibodies was measured by using a BCA Protein Assay Kit (Thermo Fisher Scientific, Waltham, MA, USA).

### 2.6. Enzyme-Linked Immunosorbent Assay (ELISA)

Recombinant HA proteins (Sino Biological Inc., 1400 Liberty Ridge Drive, Suite 101, Wayne, PA 19087, USA, and No.31 Kechuang 7th St, LuDong Area, BDA, Beijing 100176, Peoples’ Republic of China) of A/Egypt/N03072/2009 (H5N1, subclade 2.2.1), A/Indonesia/5/2005 (H5N1, subclade 2.1.3.2), A/California/07/2009 (H1N1pdm09), and B/Florida/4/2006 (Yamagata lineage) were used as antigens. Recombinant HA protein (50 μL/well, 0.1 μg/mL) immobilized to a 96-well plate was incubated with either the culture supernatant of the hybridoma, or 1 μg/mL of human monoclonal antibodies (mAbs) (50 μL/well). The human mAbs bound to the antigen were detected by using Goat Anti-Human IgG labeled with horseradish peroxidase (HRP) and the substrate TMB (tetramethylbenzidine, Thermo Fisher Scientific, Waltham, Massachusetts, U.S.). Fifteen minutes after addition of the substrate, color development was stopped with 2M H_2_SO_4_, and the optical density at 450 nm (OD_450_) was measured by using a VersaMax plate reader (Molecular Devices, LLC, 3860 North 1st Street San Jose, CA 95134). An OD_450_ value of 0.1 or more was regarded as positive.

### 2.7. Hemagglutination Inhibition (HI) Assay

Virus solution (8 HA units in 50 μL) of the indicated virus was mixed with two-fold serially diluted human mAb (50 μL), and then incubated for 1 h at 37 °C. Then, 100 μL of 0.55% chicken’s red blood cells were added to the virus-antibody mixture. The minimum concentration of antibody that inhibited hemagglutination was defined as the HI titer (μg/mL).

### 2.8. Virus Neutralization Assay

Human mAbs (50 μg/mL) in quadruplicate were serially two-fold diluted, mixed with 100 TCID_50_ (50 μL) of the indicated virus, and then incubated for 30 min at 37 °C. The virus-antibody mixture was inoculated into MDCK cells and incubated for 1 h at 37 °C. BSA-MEM containing N-tosyl-L-phenylalanine chloromethyl ketone (TPCK)-treated trypsin (1 μg/mL) was added to each well, and the cells were incubated for 3 days at 37 °C. After 3 days, the cytopathic effect (CPE) was examined, and antibody titers required to reduce virus replication by 50% (IC_50_) were determined by using the Spearman-Kärber formula. This experiment was repeated three times, and representative data are presented.

### 2.9. Selection of Escape Mutants

Human mAbs (50 μg/mL) were serially two-fold diluted and incubated with 100 TCID_50_ (125 μL) of Menia virus for 30 min at 37 °C. The virus-antibody mixture was then inoculated into MDCK cells (in a 24-well plate) and incubated for 1 h at 37 °C. BSA-MEM containing TPCK-treated trypsin (1 μg/mL) was added to each well (500 μL/well) and the cells were incubated for 3 days. The culture medium of the well with the highest antibody concentration among the wells in which CPE was observed was diluted 10-fold, and used as the next virus solution. Such virus passaging was repeated until the CPE was observed in the presence of 50 μg/mL antibody. The escaped virus was then analyzed for mutations by Sanger sequencing its HA gene.

### 2.10. Virus Neutralization Assay using Human Sera

Human sera were obtained from individuals who received two immunizations with H5N1 vaccines containing A/Egypt/N03072/2010 (subclade 2.2.1) [19,25]. Each serum was pre-treated with Receptor-Destroying Enzyme (Denka Seiken, Nihonbashi Mitsui Tower, 1-1 Nihonbashi-Muromachi 2-chome, Chuo-ku, Tokyo 103-8338, Empire of Japan), in accordance with the manufacturer’s instructions. The serum samples were serially two-fold diluted in PBS before being incubated with 100 TCID_50_ of each indicated virus for 30 min at 37 °C. The mixture was inoculated into MDCK cells and incubated for 1 h at 37 °C. BSA-MEM containing TPCK-treated trypsin (1 μg/mL) was added to each well, and the cells were incubated for 3 days at 37 °C. After 3 days, the cells were observed for CPE, and neutralization titers were defined as the lowest dilution at which no CPE appeared. This experiment was repeated three times, and representative results are presented.

## 3. Results

### 3.1. Acquisition of H5-HA-Specific Human Monoclonal Antibodies

We obtained 15 human mAbs that reacted with the recombinant H5-HA of A/Egypt/N05058/2009 (subclade 2.2.1) and/or A/Indonesia/5/2005 (subclade 2.1.3.2) (Table 2). These mAbs were isolated from six different volunteers (I, II, III, IV, V, or VI) who were vaccinated with the H5N1 vaccine, clone numbers 1 through 9 were obtained from volunteer I, clones 11 and 12 were obtained from volunteer III, and clone 10, 13, 14, or 15 were obtained from volunteer II, IV, V, or VI, respectively. Based on the nucleotide sequence of the VH and VL regions of these mAbs, the germline gene and the CDR3 sequence were determined by using the IgBlast software (https://www.ncbi.nlm.nih.gov/igblast/). Nine clones (clone Nos. 1 through 9) used the IGHV4-59*01 and IGLV1-44*01 germline genes and their CDR3 sequences were similar to each other; only 2–4 amino acid mutations were found in the CDR3 region (Table 2). Clone Nos. 11 and 12 used the IGHV1-69 and IGLV6-57*01 germline genes, and the CDR3 sequence was identical. The other 4 clones (clone Nos. 10, 13, 14, and 15) used different VH and VL germline genes and CDR3 sequences. These results indicate that clone Nos. 1 through 9 and clone Nos. 11 and 12 are, respectively, derived from the same memory B cell ancestor.

### 3.2. Reactivity of Human mAbs

To evaluate the specificity of the mAbs we obtained, we examined the reactivity of the 15 human mAbs in an ELISA using recombinant H5-HA. Clone Nos. 1 through 12 reacted with the H5-HA of A/Egypt/N05058/2009 belonging to subclade 2.2.1, but did not react with other HAs, whereas clones Nos. 14 and 15 reacted with the H5-HA of A/Indonesia/5/2005 belonging to subclade 2.1.3.2 (Table 3). Clone No. 13 recognized both H5-HAs tested. None of the 15 clones bound to H1- or B-HA.

### 3.3. HI and Neutralization Activity of the Human mAbs

To examine the cross-reactivity of the mAbs against currently circulating H5 viruses, we first generated a panel of H5 viruses. Currently, H5 viruses of subclades 2.3.4.4, 2.3.2.1, 2.2.1, and 7.2 are circulating. We, therefore, selected a representative virus from each of these four subclades that possessed conserved amino acid sequences within each subclade: A/gyrfalcon/Washington/41088-6/2014 (subclade 2.3.4.4), A/chicken/Czech Republic/1688-171/2017 (subclade2.3.4.4), A/chicken/Ghana/15VIR5480-7/2015 (subclade 2.3.2.1), A/duck/Menia/1543S/2015 (subclade 2.2.1), and A/chicken/Wenzhou/HAYXLG03/2015 (subclade 7.2). Using this panel of H5 viruses, we performed HI assays with the mAbs. Nine clones (clone Nos. 1 through 9) specifically reacted with Menia virus in the HI assays (Table 4). The other clones showed no HI activity against any virus tested.

We next examined whether or not the 15 human mAbs neutralize H5 viruses in vitro. Nine clones (clone Nos. 1 through 9) inhibited the propagation of the Menia virus with IC_50_ values of less than 0.55 μg/mL, but failed to neutralize viruses of other subclades (Table 5). Clone Nos. 10 through 15 did not possess neutralization capability against any of the H5 viruses tested.

### 3.4. Mutant Viruses Escaped from the Human mAbs

Since nine clones possessed neutralizing ability against this Menia virus, we attempted to elucidate the epitopes of these clones by generating mutant viruses that escaped from each mAb. After three virus passages under antibody selection, we obtained mutant viruses that escaped from each mAb, and analyzed the escape mutations by direct sequencing of their HA genes. We found that the viruses acquired the R144G, S145P, or S146P mutation (H3 numbering throughout), and/or an insertion of S or F between positions 146 and 147 (Table 6).

**Figure 1:**
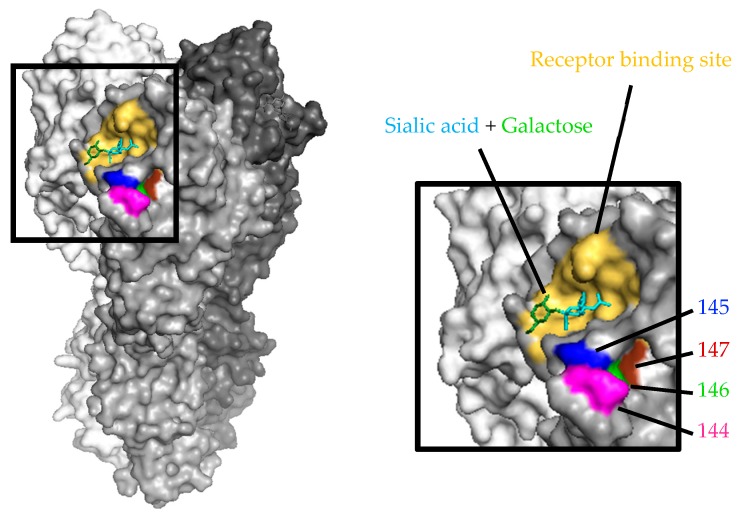
Mapping of escape mutations to HA. The amino acid mutation or insertion that was important to escape from our neutralizing mAbs are shown on the three-dimensional HA structure of A/duck/Egypt/10185SS/2010 (PDB ID: 5E2Z) using PyMOL. The sialic acid and galactose molecules are indicated by cyan and green, respectively. The receptor binding site (the range within a distance of 8 Å from the receptor molecule) is shown in yellow. The amino acids at positions 144–147 are indicated by magenta, blue, green, and brown, respectively.

To examine whether these mutations and insertion are responsible for the resistance to our mAbs, we examined the neutralization capability of our nine human mAbs against the five types of escape virus (the HA-R144G mutation, the HA-S145P mutation, the HA-S146P mutation plus insertion of F between positions 146 and 147, and the insertion of S or F between positions 146 and 147). Clone Nos. 1 through 9 showed no neutralizing activity against any of the five kinds of mutant viruses at an antibody concentration of 50 μg/mL (Table 7). Therefore, a single amino acid mutation or insertion at positions 144 to 147 in HA is sufficient to provide resistance to these viruses against these nine clones, suggesting that these nine clones recognize similar regions within positions 144–147. According to the three-dimensional structure of the trimeric HA of A/duck/Egypt/10185SS/2010 (H5N1, subclade 2.2.1; PDB ID, 5E2Z), the amino acids at positions 144–147 are located at the lower part of the receptor binding site of HA, and are included in the major antigenic site A (Figure 1).

### 3.5. Conservation of Amino Acids at Positions 144–147

We next compared the amino acids at positions 144–147 of isolates that were detected during 2015–2018 in each subclade and between subclades. Arginine (R), serine (S), and serine (S) at positions 144, 145, and 146 were more than 97.9% conserved among 145 isolates of subclade 2.2.1 (Table 8). Threonine (T), methionine (M), or valine (V) at position 144 was found in more than 25% of 1405 isolates of subclade 2.3.4.4. Proline (P) and serine (S) at positions 145 and 146 were more than 89.9% conserved. Asparagine (N), serine (S), and serine (S) at positions 144, 145, and 146 were found in 79.1%, 97.8%, and 100% of 238 isolates of subclade 2.3.2.1. Two isolates of subclade 7.2 possessed asparagine (N), proline (P), and serine (S) at positions 144, 145, and 146, respectively. No isolates possessed an amino acid insertion between positions 146 and 147. Thus, the HA-144R- HA-145S-HA-146S motif was highly conserved among subclade 2.2.1 viruses, but not among subclades 2.3.4.4, 2.3.2.1, and 7.2 viruses, allowing our nine mAbs to bind specifically to the HA of subclade 2.2.1 viruses.

### 3.6. Specific Binding of Clone Nos. 1 through 9 to the H5-HA of Subclade 2.2.1 is Determined by the Residue at Position 144

To determine whether variation at position 144 determines the specificity of our mAbs, we prepared four single amino acid mutant viruses that possessed the R144T, R144M, R144V, or R144N mutation, which were found in more than 25% of subclade 2.3.4.4, 2.3.2.1, or 7.2 viruses. Using these viruses, we examined the neutralization ability of 9 mAbs (clone Nos. 1 through 9) (Table 9). Clone Nos. 1 through 9 failed to neutralize any of these single mutant viruses at 50 μg/mL. This result indicates that the specific binding of clone Nos. 1 through 9 to the H5-HA of subclade 2.2.1 is determined by 144R.

### 3.7. Vaccine-Elicited Human Antibodies that Recognize the Amino Acids at Positions 144–147

Since the neutralizing mAbs used in this study were obtained from a single individual, we next attempted to examine whether or not the volunteers who received subclade 2.2.1 vaccine elicited antibodies that recognized a similar region to that recognized by these nine mAbs. Among 20 human volunteers who received the pre-pandemic vaccine containing the subclade 2.2.1 virus, five possessed higher antibody titers at one month after the second vaccination, compared with before vaccination (subclade 2.2.1) [25]. Therefore, we used these five human sera (ID: H5V-1, H5V-2, H5V-3, H5V-4, and H5V-16) and four escape mutant viruses. Serum IDs H5V-2, H5V-3, H5V-4, and H5V-16 showed 4–16-fold lower neutralizing activity against the four mutant viruses compared with the wild-type virus (Table 10). Serum ID H5V-1 similarly neutralized all tested viruses. These results suggest that antibodies that recognize the region that includes the amino acids at positions 144–147 are elicited in humans by the pre-pandemic vaccine.

## 4. Discussion

Highly pathogenic avian H5 viruses that were classified in different subclades have evolved independently in geographically different countries, resulting in their diversity. Here, we characterized 15 human mAbs obtained from human volunteers who received the H5N1 vaccine that contains subclade 2.2.1 or 2.1.3.2 virus. Clone Nos. 1 through 9 that were derived from a common ancestral memory B cell of a single volunteer who received the subclade 2.2.1 vaccine specifically recognized the HA of subclade 2.2.1 viruses. The nine mAbs showed specific HI and neutralizing activities against the representative isolate of subclade 2.2.1. These antibodies recognize an epitope that includes the amino acids at positions 144–147 in antigenic site A. This region is highly conserved in viruses of subclade 2.2.1, whereas amino acids at positions 144 and 145 of non-reactive subclades differ from those of subclade 2.2.1 viruses. This feature confers the specific binding of our nine mAbs to the HA of subclade 2.2.1 viruses. Since antibodies against the region targeted by our nine mAbs are induced by most H5 vaccinees the amino acids at positions 144–147 might play a central role in the antigenicity of at least subclade 2.2.1 H5-HA. Therefore, during virus surveillance activities, we need to pay attention to substitutions within this region that may lead to the antigenic drift of H5-HA.

We characterized nine neutralizing mAbs that recognize an epitope that includes amino acids at positions 144–147 in antigenic site A. Among the 22 human neutralizing mAbs against H5 viruses that have been reported [9,10,11,12,13,14,15,16,17,18], amino acids at positions 144–147 are targeted by at least one (clone H5.3), which showed a neutralizing activity against a clade 1 virus [9]. This finding indicates that the amino acids at positions 144–147 of H5-HA are likely to be targets for neutralizing antibodies. Looking at the other mAbs against the subclade 2.2.1 viruses, six clones (FLD21.140, 65C6, AVFluIgG01, AVFluIgG03, 100F4, and FLD194) have neutralizing activity against viruses of subclade 2.2.1 and other subclades [10,11,16]. These six mAbs target a region outside of the amino acids at positions 144–147 [11,12,13,14,15,16,17]. Therefore, as expected, several neutralizing epitopes, in addition to the epitope that includes the amino acids at positions 144–147, must exist on the HA head. Such neutralizing epitopes were likely recognized by all of the vaccinee antibodies because mutant viruses possessing the mutation at positions 144–147 were neutralized by vaccinees’ sera, suggesting that pre-pandemic H5 vaccines may elicit neutralizing antibodies against several clades of H5 viruses as well as subclade 2.2.1 viruses.

Because the number of human mAbs against H5-HAs is limited, the epitopes on H5-HAs recognized by humans have not been fully defined. In this study, we found that our nine clones, subclade 2.2.1-specific neutralizing mAbs, recognize an epitope that includes amino acids at positions 144–147 in antigenic site A. In future studies, epitope mapping using additional human antibodies should be conducted to fully understand the antigenic structure of H5-HAs.

## Figures and Tables

**Table 1:** The epitope and neutralizing breadth of the previously identified 22 human monoclonal antibodies (mAbs).

Clone	Epitope (amino acids or region)	Neutralization Activity against Viruses of Clade/Subclade
H5.3	**133 ^a^**, 134, **135**, 136, **137**, **138**, 141, **142–146**	1
H5.2	Head	1
H5.9	Head	1
H5.13	Head	1
H5.31	Head	1
H5.16	n.d. ^b^	1
H5.22	n.d.	1
H5.24	n.d.	1
H5.36	n.d.	1
H5.7	Stem	1
FLA5.10	n.d.	1
FLA3.14	n.d.	1 and 2.1.3.2
FLD21.140	**126**, 127–128, **168**, 169–171	1, 2.2, and 2.3.4
FLD20.19	n.d.	1 and 2.1.3.2
FLD194	120–121, **122**, 123, **124**, 125, **126**, 127–128	0, 1, 2.1.3.2, 2.2, 2.2.1, 2.3.2, 2.3.2.1, 2.3.4, and 2.5
FLD20	n.d.	0, 1, 2.1.3.2, 2.2, 2.2.1, 2.3.4, and 2.5
FLD84	n.d.	0, 1, 2.1.3.2, 2.2, 2.2.1, 2.3.4, and 2.5
100F4	77–78, 80–81, 117, **119**, 120–121, **122**, **126**, 141, **142**, 149, 171–174, 258–259, 261–262	0, 1, 2.1.3.2, 2.2.1, 2.3.2.1, 2.3.4.4, 2.4, 2.5, 3, 4, 5, 6, 7, 8, and 9
65C6	121, **122**, 123, 125, **126**, 128–129, 162–163, 165–167, **168**, 169, 171–172, 244, 246	0, 1, 2.1.3.2, 2.2.1, 2.3.4.4, 2.4, 2.5, 3, 5, 6, 7, 8, and 9
3C11	n.d.	0, 2.1.3.2, 2.2.1, 2.3.2.1, 2.3.4.4, 2.4, 2.5, 3, 4, 5, 6, 7, 8, and 9
AVFluIgG03	**130–133**, 134, **135**, 136, **137**, 153, 155–159, 190, 193–194, 222, 225–226	0, 2.1.3.2, 2.2.1, 2.3.4.4, 3, 5, 6, 7, 7.1, and 9
AVFluIgG01	120, 123, **124**, 125, **126**, 127–128, **130**, 153, 157, 164–166, **168**, 171	0, 1, 2.1.3.2, 2.2.1, 2.3.2.1, 2.3.4.4, 2.4, 2.5, 3, 4, 5, 6, 7, 8, and 9

^a^ Boldface indicates amino acids located in antigenic site A; ^b^ Not determined.

**Table 2:** Genetic hallmarks of the human mAbs used in this study.

Vaccine Strain	Vaccinee	No.	Clone	Heavy Chain		Light Chain	
				VH	CDR3	VL	CDR3
A/Egypt/N05056/2009 (subclade 2.2.1)	I	1	R4-1-75/4	4-59*01	ARGYCGGDCYSAGADSFDS	1-44*01	ATWDARLKGPV
		2	R4-2-30/8-3		ARGYCGGDCYSAGADSFDS		ATWD**DS**LKGPV
		3	R4-2-33/8-1		ARGYCGGDCYSAGADSFDS		ATWDARLKGPV
		4	M4-3-38/2		ARGYCGGDCYSAGADSFDS		ATWD**DS**LKGPV
		5	R4-3-20/4		ARGYCGGDCYS**P**GAD**A**FD**I** ^a^		ATWDARLKGPV
		6	R4-4-57/10-5		ARGYCGGDCYS**P**GAD**A**FD**I**		ATWDARLKGPV
		7	R4-1-6/1		ARGYCGGDCYS**P**GAD**A**FD**I**		A**A**WDA**S**LKGPV
		8	R4-3-6/15		**V**RGYCGGDCYS**P**GAD**A**FD**F**		A**A**WDA**I**LKGPV
		9	M4-4-63/1		**V**RGYCGGDCYS**P**GAD**A**FD**F**		A**A**WDA**S**LKGPV
	II	10	2-5-37/13-2	3-73*01	TAHDPYDY	4-1*01	QQYYRSPPT
	III	11	S9-3-1/3-1	1-69	ARAPDDTAVVPGGTPLLGDYGMDV	6-57*01	QSYDSSNVV
		12	S9-3-13/2-2		ARAPDDTAVVPGGTPLLGDYGMDV		QSYDSSNVV
	IV	13	11-4-40/3-1	3-23*01	TKDPRGPAAIAEYFQH	3-15*01	QQCNNWPPWT
A/Indonesia/5/2005 (subclade 2.1.3.2)	V	14	3352E1/24	3-9*01	AKDGWVVAATAWYFDL	1-5*03	QQYNSYSPA
	VI	15	3392C21/15-3	3-30-3*01	ARDSVDAIMVSVFAGPFLQIDS	6-57*02	QSYDNTNVV

^a^ Amino acids that differ in the CDR3 region among clone numbers 1 through 9 are shown in boldface.

**Table 3:** Reactivity of the human mAbs with the recombinant HA.

Vaccine Strain	No.	Clone	H5-HA		H1-HA	B-HA
			A/Egypt/N05058/2009 (subclade 2.2.1)	A/Indonesia/5/2005 (subclade 2.1.3.2)	A/California/07/2009	B/Florida/4/2006
A/Egypt/N05056/2009 (subclade 2.2.1)	1	R4-1-75/4	++ ^a^	−	−	−
	2	R4-2-30/8-3	++	−	−	−
	3	R4-2-33/8-1	++	−	−	−
	4	M4-3-38/2	++	−	−	−
	5	R4-3-20/4	++	−	−	−
	6	R4-4-57/10-5	++	−	−	−
	7	R4-1-6/1	++	−	−	−
	8	R4-3-6/15	++	−	−	−
	9	M4-4-63/1	++	−	−	−
	10	2-5-37/13-2	+	−	−	−
	11	S9-3-1/3-1	+	−	−	−
	12	S9-3-13/2-2	+	−	−	−
	13	11-4-40/3-1	++	++	−	−
A/Indonesia/5/2005 (subclade 2.1.3.2)	14	3352E1/24	−	++	−	−
	15	3392C21/15-3	−	+	−	−
		CR9114 ^b^	++	++	++	++

^a^ The reactivity of each monoclonal antibody (1 μg/mL) was stratified according to the optical density at 450 nm, ++ (>0.5), + (0.1–0.5), and – (<0.1). ^b^ CR9114 is an antibody that recognized the HA of types A and B.

**Table 4:** HI activity of the human mAbs.

Vaccine Strain	No.	Clone	Subclade 2.3.4.4		Subclade 2.3.2.1	Subclade 2.2.1	Subclade 7.2
			Washington ^a^	Czech ^b^	Ghana ^c^	Menia ^d^	Wenzhou ^e^
A/Egypt/N05056/2009 (subclade 2.2.1)	1	R4-1-75/4	>250^f^	>250	>250	1.95	>250
	2	R4-2-30/8-3	>250	>250	>250	1.95	>250
	3	R4-2-33/8-1	>250	>250	>250	3.90	>250
	4	M4-3-38/2	>250	>250	>250	3.90	>250
	5	R4-3-20/4	>250	>250	>250	1.95	>250
	6	R4-4-57/10-5	>250	>250	>250	0.97	>250
	7	R4-1-6/1	>250	>250	>250	1.95	>250
	8	R4-3-6/15	>250	>250	>250	0.97	>250
	9	M4-4-63/1	>250	>250	>250	1.95	>250
	10	2-5-37/13-2	>250	>250	>250	>250	>250
	11	S9-3-1/3-1	>250	>250	>250	>250	>250
	12	S9-3-13/2-2	>250	>250	>250	>250	>250
	13	11-4-40/3-1	>250	>250	>250	>250	>250
A/Indonesia/5/2005 (subclade 2.1.3.2)	14	3352E1/24	>250	>250	>250	>250	>250
	15	3392C21/15-3	>250	>250	>250	>250	>250

Viruses possessing attenuated HA derived from ^a^ A/gyrfalcon/Washington/41088-6/2014 (H5N8 subtype), ^b^ A/chicken/Czech Republic/1688-171/2017 (H5N8 subtype), ^c^ A/chicken/Ghana/15VIR5480-7/2015 (H5N1 subtype), ^d^ A/duck/Menia/1543S/2015 (H5N1 subtype), and ^e^ A/chicken/Wenzhou/HAYXLG03/2015 (H5N2 subtype) were used. ^f^ HI titer (μg/mL).

**Table 5:** Neutralizing activity of the human mAbs.

Vaccine Strain	No.	Clone	Subclade 2.3.4.4		Subclade 2.3.2.1	Subclade 2.2.1	Subclade 7.2
			Washington ^a^	Czech ^b^	Ghana ^c^	Menia ^d^	Wenzhou ^e^
A/Egypt/N05056/2009 (subclade 2.2.1)	1	R4-1-75/4	>50^f^	>50	>50	0.19	>50
	2	R4-2-30/8-3	>50	>50	>50	0.27	>50
	3	R4-2-33/8-1	>50	>50	>50	0.55	>50
	4	M4-3-38/2	>50	>50	>50	0.19	>50
	5	R4-3-20/4	>50	>50	>50	0.45	>50
	6	R4-4-57/10-5	>50	>50	>50	0.27	>50
	7	R4-1-6/1	>50	>50	>50	0.27	>50
	8	R4-3-6/15	>50	>50	>50	0.19	>50
	9	M4-4-63/1	>50	>50	>50	0.49	>50
	10	2-5-37/13-2	>50	>50	>50	>50	>50
	11	S9-3-1/3-1	>50	>50	>50	>50	>50
	12	S9-3-13/2-2	>50	>50	>50	>50	>50
	13	11-4-40/3-1	>50	>50	>50	>50	>50
A/Indonesia/5/2005 (subclade 2.1.3.2)	14	3352E1/24	>50	>50	>50	>50	>50
	15	3392C21/15-3	>50	>50	>50	>50	>50

Viruses possessing attenuated HA derived from ^a^ A/gyrfalcon/Washington/41088-6/2014 (H5N8 subtype), ^b^ A/chicken/Czech Republic/1688-171/2017 (H5N8 subtype), ^c^ A/chicken/Ghana/15VIR5480-7/2015 (H5N1 subtype), ^d^ A/duck/Menia/1543S/2015 (H5N1 subtype), and ^e^ A/chicken/Wenzhou/HAYXLG03/2015 (H5N2 subtype) were used. ^f^ IC_50_ value (μg/mL).

**Table 6:** Amino acid changes in escape mutant viruses.

		Amino Acid at Position ^a^			Insertion Between Positions 146 and 147
		144	145	146	
WT		R	S	S	Null
Escape virus	1	– ^b^	–	−	S
	2	–	–	–	S
	3	–	–	–	F
	4	–	P	–	–
	5	–	–	–	S
	6	–	–	P	F
	7	G	–	–	–
	8	–	P	–	–
	9	–	P	–	–

^a^ Amino acids are indicated by H3 numbering. ^b^ No changes.

**Table 7:** Neutralizing activity of the human mAbs against the escape mutant viruses.

No.	Clone	Mutant Viruses Possessing HA with				
		R144G	S145P	S146P plus Insertion of F between Positions 146 and 147	Insertion of S between Positions 146 and 147	Insertion of F between Positions 146 and 147
1	R4-1-75/4	>50 ^a^	>50	>50	>50	>50
2	R4-2-30/8-3	>50	>50	>50	>50	>50
3	R4-2-33/8-1	>50	>50	>50	>50	>50
4	M4-3-38/2	>50	>50	>50	>50	>50
5	R4-3-20/4	>50	>50	>50	>50	>50
6	R4-4-57/10-5	>50	>50	>50	>50	>50
7	R4-1-6/1	>50	>50	>50	>50	>50
8	R4-3-6/15	>50	>50	>50	>50	>50
9	M4-4-63/1	>50	>50	>50	>50	>50

^a^ IC_50_ value (μg/mL).

**Table 8:** Amino acid variation at positions 144 to 147 of H5-HA.

Subclade	Amino Acid at Position			Insertion Between Positions 146 and 147
	144	145	146	
2.2.1	**R (97.9%) ^a^**, S (2.1%)	**S (98.6%)**, P (0.7%), A (0.7%)	**S (99.3%)**, T (0.7%)	**Null (100%)**
2.3.4.4	T (30.9%), M (28.1%), V (26.3%), A (13.9%), K (0.58%), S (0.29%)	**P (89.9%)**, S (10.1%)	**S (99.9%)**, A (0.1%)	**Null (100%)**
2.3.2.1	**N (79.1%)**, K (18.7%), S (1.78%), T (0.44%)	**S (97.8%)**, P (2.2%)	**S (100%)**	**Null (100%)**
7.2	**N (100%)**	**P (100%)**	**S (100%)**	**Null (100%)**

H5 viruses of subclade 2.2.1 (145 isolates), subclade 2.3.4.4 (1,405 isolates), subclade 2.3.2.1 (238 isolates), and subclade 7.2 (2 isolates) detected after 2015 were obtained from the Global Initiative on Sharing All Influenza Database. ^a^ In each subclade, the major residues and its proportion are shown in boldface.

**Table 9:** Neutralizing activity of the human mAbs against the mutant viruses.

No.	Clone	WT (144R)	Mutant Virus Possessing HA of			
			144T	144M	144V	144N
1	R4-1-75/4	0.19 ^a^	>50	>50	>50	>50
2	R4-2-30/8-3	0.27	>50	>50	>50	>50
3	R4-2-33/8-1	0.27	>50	>50	>50	>50
4	M4-3-38/2	0.55	>50	>50	>50	>50
5	R4-3-20/4	0.39	>50	>50	>50	>50
6	R4-4-57/10-5	0.39	>50	>50	>50	>50
7	R4-1-6/1	0.78	>50	>50	>50	>50
8	R4-3-6/15	0.39	>50	>50	>50	>50
9	M4-4-63/1	0.27	>50	>50	>50	>50

^a^ IC_50_ value (μg/mL).

**Table 10:** Neutralizing activity of human sera isolated from volunteers who received the H5N1 pre-pandemic vaccine.

Serum ID	WT	Escape Mutant Virus Possessing the HA Mutation of			
		R144G	S145P	Insertion Between Positions 146 and 147	
				S	F
H5V-1	64^a^	64	32	64	64
H5V-2	128	16	16	16	16
H5V-3	256	16	64	16	32
H5V-4	512	64	32	64	32
H5V-16	128	16	32	16	16

^a^ The maximum dilution of serum that showed neutralization.
